# Target capture sequencing reveals a monoclonal outbreak of respiratory syncytial virus B infections among adult hematologic patients

**DOI:** 10.1186/s13756-022-01120-z

**Published:** 2022-06-21

**Authors:** Claas Baier, Jiabin Huang, Kerstin Reumann, Daniela Indenbirken, Felicitas Thol, Christian Koenecke, Ella Ebadi, Albert Heim, Franz-Christoph Bange, Sibylle Haid, Thomas Pietschmann, Nicole Fischer

**Affiliations:** 1 Institute for Medical Microbiology and Hospital Epidemiology, Hannover Medical School (MHH), Carl-Neuberg-Straße 1, 30625 Hannover, Germany; 2 Institute for Medical Microbiology, Virology and Hygiene, University Medical Center Hamburg-Eppendorf, Martinistraße 52, 20246 Hamburg, Germany; 3 Leibniz Institute for Experimental Virology, Martinistraße 52, 20251 Hamburg, Germany; 4 Department of Hematology, Hemostasis, Oncology and Stem Cell Transplantation, Hannover Medical School (MHH), Carl-Neuberg-Straße 1, 30625 Hannover, Germany; 5 Institute of Virology, Hannover Medical School (MHH), Carl-Neuberg-Str. 1, 30625 Hannover, Germany; 6 Institute for Experimental Virology; Twincore-Centre for Experimental and Clinical Infection Research; a joint venture of Hannover Medical School (MHH) and Helmholtz Centre for Infection Research (HZI), Feodor-Lynen-Straße 7, 30625 Hannover, Germany; 7 Cluster of Excellence RESIST (EXC 2155), Hannover Medical School, Carl-Neuberg-Straße 1, 30625 Hannover, Germany; 8 German Center for Infection Research (DZIF), Partner Site Hannover-Braunschweig, 30625 Hannover, Germany

**Keywords:** Respiratory syncytial virus, Outbreak, Hematology, Infection, Infection control, Capture probe sequencing, Molecular epidemiology

## Abstract

**Background:**

Respiratory syncytial virus (RSV) causes community-acquired respiratory tract infections during winter. However, outbreaks in hospitals also occur repeatedly. In particular, patients with hematologic malignancies are at an increased risk for a severe and potentially fatal course of RSV infection. Here we present the investigation of an RSV outbreak in a hematology ward for adults following the ORION statement.

**Methods:**

An epidemiologic and molecular outbreak analysis was performed. We developed and employed a minimal oligonucleotide probe set in target capture probe sequencing that allows cost-effective RSV-A or -B capturing to reconstruct RSV genomes from clinical samples.

**Results:**

Four adult patients were involved in the outbreak caused by RSV-B in March 2019. The enforcement of the pre-existing infection control measures by effective training of hospital staff contributed to a successful containment. PCR-based RSV screening on the ward enabled early detection of new cases and rapid isolation measures. The molecular analysis demonstrated that the outbreak sequences were highly related and distinct to other RSV-B strains circulating at the same time.

**Conclusions:**

A multimodal infection control concept is essential for the timely detection and control of RSV outbreaks in patients with hematological disease. Among other measures, preventive screening for respiratory viruses is recommended. Furthermore, the integration of conventional and molecular epidemiology, such as whole-genome sequencing and variant calling, significantly contributes to the understanding of transmission pathways. Based on this, appropriate conclusions can be drawn for targeted prevention measures that have prepared us for the COVID-19 pandemic beyond the RSV approach described here.

**Supplementary Information:**

The online version contains supplementary material available at 10.1186/s13756-022-01120-z.

## Background

Respiratory syncytial virus (RSV) is an enveloped RNA virus. It is responsible for infections of the upper (URTI) and lower respiratory tract (LRTI) in a seasonal rhythm in countries with temperate climates [1–4]. The pathogen is primarily transmitted by droplets from the respiratory tract and less often by contact. Aerosol transmission might play a role under certain circumstances as well [5]. The median incubation period is reported to be 4 to 5 days [6]. RSV outbreaks in hematology and oncology units (e.g., [7–10]) and other hospital settings [11] have been repeatedly reported in the last years. For infection control of RSV in healthcare facilities, several measures (e.g., isolation, personal protective equipment) are usually combined to prevent nosocomial spread [12]. Patients with a hematologic malignancy are at high risk for a severe RSV LRTI [13–16]. Especially, leukopenia seems to be a relevant risk factor for increased mortality [17].

Herein, we report the epidemiology, control and molecular investigation of a hospital outbreak caused by RSV-B affecting 4 adults on a hematology ward. In addition, we discuss how the existing infection control concept for RSV in hematology helped us in the coronavirus disease 2019 (COVID-19) pandemic.

## Methods

### Epidemiologic analysis

The epidemiologic analysis followed the principles of the ORION statement [18]. Patient charts, virology results and patient movement data were reviewed for data acquisition. In addition, a timeline was generated.

### Case definition

An outbreak case was a patient from the affected ward with nosocomial acquisition of RSV in March 2019. Nosocomial acquisition was assumed when RSV was found on day 5 or later of the patient’s stay on the ward.

### Routine infection control measures for RSV

The infection control concept at our institution for hematology wards regarding RSV is summarized in Table 1. It was primarily shaped by the management of a previous RSV outbreak in a pediatric setting in our institution [10] and included a weekly PCR prevalence screening for asymptomatic patients [19]. This screening also addressed influenza.

**Table 1:** Basic infection control measures regarding RSV at the hematology ward

**Isolation of RSV positive patients:**
RSV positive patients were isolated (single room or grouping of patients, if more than one RSV patient was present). Isolation was kept until at least one respiratory specimen was tested negative by PCR or had a ct value > = 35 and the patient’s medical condition improved
**Prophylactic isolation of patients with respiratory symptoms:**
Newly admitted patients with respiratory symptoms and already hospitalized patients with onset of respiratory symptoms were separated from other patients and tested for a panel of respiratory viruses
**RSV specific contact and droplet precautions:**
Visitors and HCWs wore a surgical mask, a gown and gloves whenever entering the room of a RSV positive patient. RSV infected patients were asked to stay preferably in their individual room and were instructed in hand hygiene. Outside of the room RSV positive patients wore surgical masks (e.g., during urgently necessary examinations)
**Quarantine:**
Other patients sharing a room with a patient who tested positive for RSV were put in quarantine for 8 days
**Preemptive droplet precautions on the ward (universal masking):**
All HCWs and visitors wore surgical masks at any time when on the ward during winter season (usually December to March). The same applied to all patients when they left their patient room. Visitors were intensively instructed in droplet precautions and hand hygiene
**Prophylactic RSV Screening*:***
Newly admitted patients were tested for RSV/Influenza (admission screening). In addition, once weekly a RSV/Influenza screening for all patients on the ward took place (prevalence screening) [19, 20]
**Pre-Season Audits/Training:**
Training for HCWs provided by the infection control staff prior to the winter season
**Protective isolation:**
Nursing in separate rooms for selected patients (e.g., for hematology patients with an expected prolonged and severe leucopenia)

### Diagnostic virology

Combined nose/throat swabs or pharyngeal washes were taken for routine viral diagnostics. If available, material from the lower respiratory tract was suitable as well. Samples were processed at the Institute of Virology of the Hannover Medical School using the reverse transcription polymerase chain reaction (RT-PCR) based Panther Fusion® System (Hologic) [20]. This system allowed a semi-quantitative diagnostic using PCR cycle threshold (ct) values.

### RNA extraction and cDNA synthesis

RNA was extracted from diagnostic samples using the Qiagen QIAamp MinElute Virus spin kit (Qiagen #57704) according to the instructions of the manufacturer. In brief, 125 µl of sample input was used and purified RNA was eluted in 50 µl water.

cDNA synthesis was performed according to the Superscript IV RT cDNA first-strand synthesis protocol (Thermo Fisher Scientific) involving RiboLock RNase Inhibitor and pdN6 random priming. 2nd strand synthesis was achieved with NEB Ultra RNA non-directional Second Strand Synthesis Module (New England Biolabs). cDNA was purified with Agencourt AMPure XP beads (Beckman Coulter Life Sciences).

### Capture probe design

The initial step of the probe design pipeline is to select the representative strains of RSV that will be included in the analysis (see Additional files 1 and 2). A total of 1101 complete genomes were downloaded from GenBank [21] and aligned using the MAFFT tool [22]. We then entered the generated alignments into the BaitsTools tool [23] for bait generation. To reduce redundancy, we clustered the bait candidates with 80% identity using usearch [24] and kept only the centroid of each cluster. Finally, we filtered out the self-complementary baits and the baits that had at least 90% identity with other baits in a blastn [25] search. To ensure the quality of the final baits, we checked all final sequences for melting temperature and GC content. The outline of the capture probe design is illustrated in Additional file 3; sequences of the capture probes are provided in Additional file 4.

### Library preparation, hybridization capture and sequencing

First, ds-cDNA was enzymatically fragmented using the SureSelect XT HS and XT Low Input Enzymatic Fragmentation Kit (Agilent). Library preparation was performed following the NEB protocol for using NEBNext DNA Ultra Library Prep Kit protocol E7370 (New England Biolabs) with the following modifications. For adaptor ligation, adaptors were diluted (1:10 or 1:25), if input cDNA was low, and USER™ enzyme volume was reduced to 2 µl. PCR cycles for library amplification were between 12 and 15 cycles depending on the amount of input cDNA.

Target capture enrichment was performed using xGen Lockdown Probes (see capture probe design) and reagents (Integrated DNA Technologies; IDT) according to the manufacturer’s instructions. Briefly, DNA capture was achieved with 500 ng barcoded library, hybridization of the probes to the library was performed for 4 h at 65 °C. Subsequently, probe/cDNA mix was bound to washed Streptavidin beads (M-270 Streptavidin beads Thermo Scientific) for 45 min at 65 °C. Stringent Streptavidin beads washing steps (stringent washing buffer) were performed at 65 °C, followed by washing steps at room temperature involving wash buffers I-III. Beads were resolved in 20 µl nuclease-free water and post-capture PCR enrichment was performed by using KAPA HiFi HotStart ReadyMix (Roche) with 2 μl NEXTFLEX ChIP Primer Mix for Illumina (PerkinElmer Applied Genomics). 20 μl captured DNA input diluted in 50 μl total volume was incubated 45 s at 98 °C, followed by 18 cycles of 15 s at 98 °C/30 s at 60 °C/30 s at 72 °C and final extension for 1 min at 72 °C. The library was purified using Agencourt AMPure XP beads (Beckman Coulter Life Sciences). Concentrations of all samples were measured with a Qubit 2.0 Fluorometer (Thermo Fisher Scientific) and fragment lengths distribution of the final libraries was analyzed with the DNA High Sensitivity Chip on an Agilent 2100 Bioanalyzer (Agilent Technologies). Sequencing was performed on the Illumina MiSeq platform (2 × 150-nt) paired reads and 3-5 Mio reads per sample.

### Bioinformatic analysis

As a result of capture probe sequencing of 4 samples from the patients (P1 – P4) involved in the outbreak and of 3 control patients with RSV infections on different wards (C1—C3, reference cohort), total numbers of 2,394,484, 2,700,477, 2,790,882, 2,384,508, 2,200,353, 2,191,289, and 1,858,890 2 × 151-nucleotide (nt) paired-end reads were generated from the Illumina MiSeq sequencer by using the designed probes, respectively. Sequence raw data were subjected to quality control using FastQC [26]. Adapter sequences of the reads and bases with a score of less than Q30 were trimmed and any reads shorter than 36 nt removed using Trimmomatic v0.36. [27]. By removing the sequence reads originating from the host organism, a set of non-host high-quality paired-end reads were prepared and fed into the SPAdes assembler (version 3.7.1), resulting in complete genomes for all patients, respectively. For each sample, we mapped the non-host reads to its newly assembled genome using Novoalign V3.07.00 (http://www.novocraft.com) with the parameters '-r Random -l 20 -g 40 -x 20 -t 100 -k', resulting in the alignment file. Then we used the tools samtools [28] and MarkDuplicate available in Picard tools (http://broadinstitute.github.io/picard/) [29] to sort the alignments and remove duplicate sequences from the alignment. The derived alignment was fed into V-Phaser2 [30] for intra-individual single nucleotide variation (iSNV) identification. In the variant identification, we only considered variants supported by at least five reads on each strand, and the ratio of the number of reads on the two strands is smaller than 10. Furthermore, the reads that mapped twice or more on the sequence were discarded. In the end, the iSNVs whose allele frequency is smaller than 0.5% were filtered out from the final results.

## Results

### Setting

The cluster took place in March 2019. The affected ward, which managed adult chemotherapy patients for hematologic malignancies and autologous hematopoietic stem cell transplantations, included 28 beds (four one-bedrooms, six two-bedrooms, and three four-bedrooms). The one-bed rooms were equipped with an anteroom and all rooms had en-suite bathrooms and high efficiency particulate air filtration. The ward was operated by qualified healthcare workers (HCWs) and housekeeping staff.

### Epidemiology

**Fig. 1:**
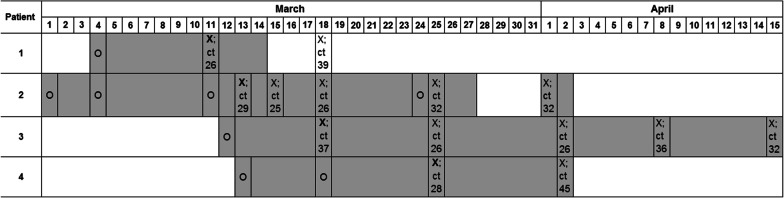
Timeline. Grey bars represent the patient’s stay on the ward. ‘X’ indicates a positive RSV testing. The first positive testing is indicated with a bold ‘**X**. ‘O’ indicates a negative testing. Ct = cycle threshold

A total of 4 patients were tested RSV positive in respiratory specimens and had been previously tested RSV negative on admission. Patient characteristics and the time course of the outbreak are shown in Table 2 and Fig. 1. Patient 1 was identified by the prevalence screening for RSV at the beginning of the second week of the hospital stay. Patient 2 was a roommate to patient 1 for several days prior to the positive test of patient 1 and was therefore tested immediately after the positive test result of patient 1 became available. Patients 3 and 4 were detected in the prevalence screening for RSV. Patient 3 shared the room for 1 to 2 h with patient 2 one day before patient 2 was tested positive. Patient 4 did not share a room with patients 1, 2 or 3 prior to the onset of RSV infection. All patients were mobile and able to leave their rooms prior to RSV positive testing.

**Table 2:** Patient’s characteristics

Patient	Nosocomial onset	Underlying disease	RSV infection	White blood cells^a^ (per microliter)	RSV Treatment	Antibiotic treatment	Oxygen need	RSV-related outcome
1	Yes	Recurrent AML	URTI	2200	Immunoglobulins	No	No	Recovered
2	Yes	Multiple myeloma	LRTI	0	Immunoglobulins	Yes	Yes	Recovered
3	Yes	Secondary hemophagocytosis	URTI	1800	None	Yes	No	Recovered
4	Yes	Multiple myeloma	URTI	0	Immunoglobulins	Yes	No	Recovered

^a^At time (± 2 days) of virus detection

### Clinical course

All involved patients were asymptomatic at the time of initial virus detection. Patient 1 quickly developed an URTI with dry coughing as the main symptom. Patient 2 developed a LRTI with oxygen need (nasal cannula) and dyspnea. This patient had a pre-existing chronic obstructive pulmonary disease and was an active smoker. Patient 3 and 4 both developed only mild RSV-associated respiratory tract infection symptoms with nasal congestion and mild coughing. No RSV-associated mortality was observed. Patients 2, 3, and 4 received antibiotics as a preventive measure against potential bacterial co-infections/superinfections. Patients 2 and 3 showed prolonged viral shedding, up to 29 days in patient 3, as demonstrated by RT-qPCR positive respiratory samples.

### Control measures

Outbreak management was initiated immediately after the second positive patient was identified. The infection control specialists enforced the pre-existing control measures (Table 1) by several ad hoc training sessions on the ward. HCWs, service personnel and visitors were re-instructed to use personal protective equipment (primarily surgical masks) and to perform hygienic hand disinfection. Moreover, we monitored hand hygiene adherence by ad hoc direct observation (compliance rate of about 70%) and gave feedback talks.

All RSV-positive tested patients were immediately isolated.

### Molecular analysis

**Fig. 2:**
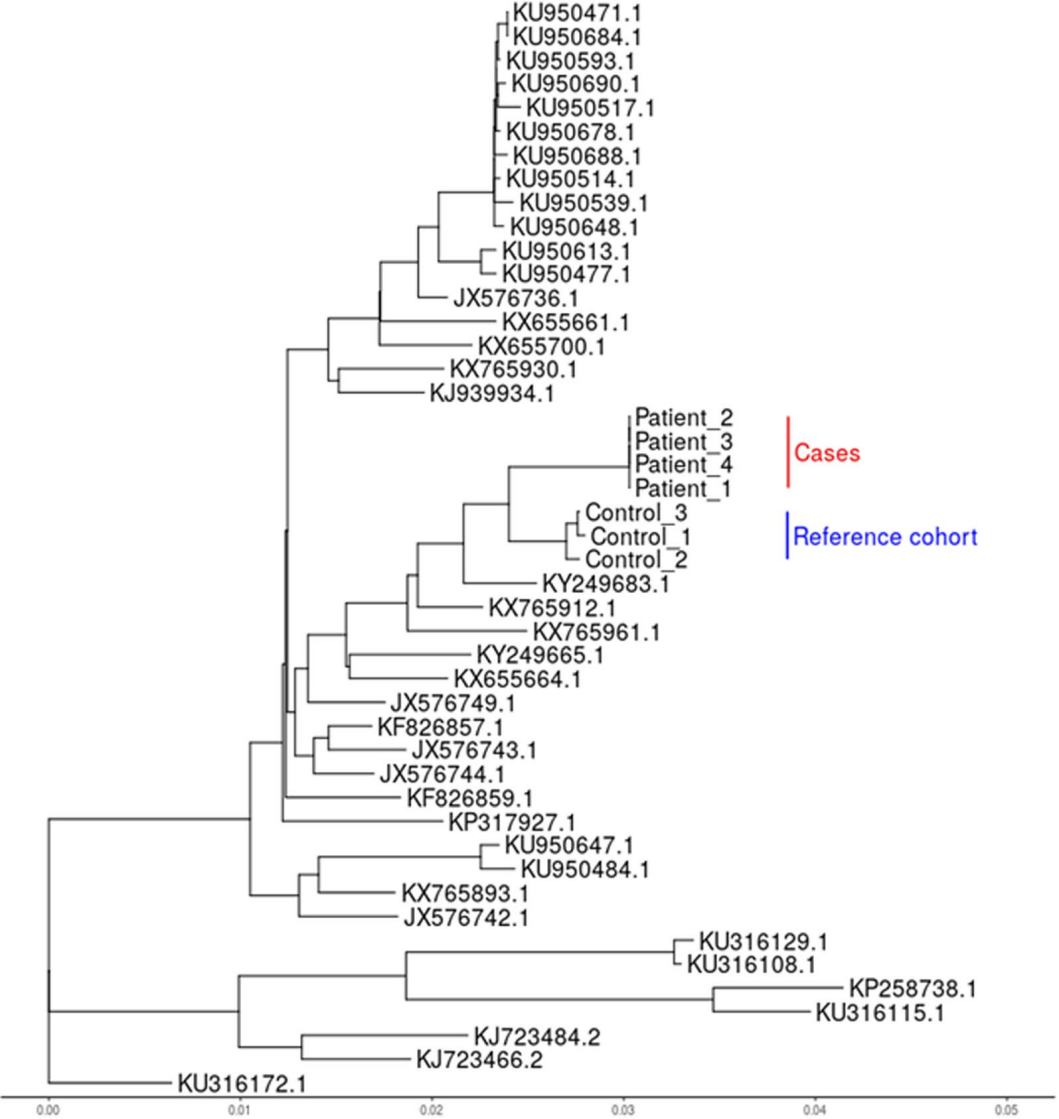
Phylogenetic analysis based on whole genome sequences of RSV isolates, subgroup B. The evolutionary history was inferred using the Maximum Likelihood method based on the General Time Reversible model. The tree with the highest log-likelihood is shown. The initial tree for the heuristic search was randomly generated. A discrete Gamma distribution was used to model evolutionary rate differences among sites with four categories. The tree is drawn to scale, with branch lengths measured in the number of substitutions per site. All positions containing gaps and missing data were eliminated

To confirm our hypothesis that all 4 patients were infected with the identical RSV strain and that these transmissions occurred on the ward, we performed RSV target capture sequencing using enrichment techniques in these 4 patients. In parallel, we examined 3 patients with RSV identified at the same time on different wards to test the hypothesis that this was not a common RSV genotype that was repeatedly introduced to the ward. These 3 control patients allowed us to assess the variability of RSV circulating in the same geographic area during this time period. We were able to determine the whole genome sequence of 15,185 nucleotides from all 7 specimens included. Detailed information on the sequencing of each genome and the coverage can be found in Additional files 5 and 6. Phylogenetic classification identified both patient and control genomes as RSV-B (Fig. 2). The RSV sequences of the 4 outbreak patients were identical to each other and had 83–87 single nucleotide polymorphisms to the RSV-B controls (see Additional file 7).

## Discussion

We investigated the epidemiologic and molecular characteristics of a RSV-B cluster affecting adult hematologic patients.

Transmission between patients, in particular patients sharing a room, is mainly conducted by droplets in RSV outbreaks [10, 31, 32]. Therefore, we assumed a direct transmission from patient 1 to patient 2 due to the shared room occupancy. The initial RSV infection source of patient 1, however, remained unclear. Patient 1 received visits and had contact to the ward’s staff for several days prior to acquisition. However, visitors and staff were urged to wear surgical masks during patient contact. Because the time interval between the positive test results of patients 1 and 2 was relatively short, a shared unknown source of infection would also be conceivable.

Interestingly, patients 2 and 3 shared for several hours one patient room before patient 2 was placed in quarantine in a single room following the positive test result of patient 1. Patient 3 tested positive 5 days later at a prevalence screening. This temporal sequence agrees well with the RSV incubation period. Thus, patient-to-patient contact could have also been the transmission route for patient 3. Patient 4 did not share a room with any other outbreak patients. Therefore, direct transmission due to co-hospitalization in the same patient room can be excluded. However, it is conceivable that there was a contact of patient 4 on the hallway or in the ward lounge with patient 3 before spatial isolation of patient 3. A random and independent occurrence of different RSV strains was implausible to us because of the clear nosocomial occurrence and the overlapping time periods. This was further confirmed by virus sequencing, which confirmed infection of the patients by the same viral strain (see below).

To our surprise, the cluster occurred although several control measures recommended for hematology wards were already in place [33, 34]. These control measures addressed potential sources (patients, healthcare workers, visitors) and transmission pathways (droplets, contact) of RSV in the hospital setting, as previously described in other outbreaks [8, 10, 31].

A core measure containing the spread by droplets is universal masking (surgical masks or FFP2/KN95). Noteworthy, this measure has been widely adopted in hospitals in the current COVID-19 pandemic and has also shown a reduction of nosocomial RSV and influenza as a side effect [35]. Starting March 2020, we used strict universal masking (staff and patients) in the COVID-19 pandemic with the hematologic ward affected by the outbreak described here being a role model for other wards.

Nursing in separate rooms of selected hematology patients (e.g., patients with an expected prolonged and severe leucopenia) is another important measure and can contribute to outbreak prevention and containment.

In our case, the outbreak control management focused explicitly on enforcing the already existing measures, thereby successfully containing the outbreak. The established PCR-based screening program (including influenza) [19] was helpful in rapid detection of the index patient and patients 3 and 4, both being asymptomatic at the time of the PCR screening. The infection control concept of prevalence and admission screening has also been widely adopted during the COVID-19 pandemic in healthcare facilities. Moreover, it might be useful to extend a screening panel to other respiratory viruses such as human metapneumovirus, which can also cause (severe) morbidity and mortality in immunocompromised hematology patients.

Integrating molecular sequence analysis with temporal and spatial information from classical epidemiology significantly increases the understanding of outbreak events. Building on this, we tested and confirmed our epidemiological hypothesis regarding the chain of transmission by target capture sequencing. The RSV-B isolates from the 4 patients were identical but significantly distinct from other RSV-B isolates found during the same time period in our institution. Given the molecular and epidemiologic analysis, we assume that patient-to-patient transmission among patients 1 to 3 is likely and that patient 4 is part of the outbreak as well. The transmission route in the latter patient, however, remains unclear. Initial introduction of the RSV outbreak strain by a point source (e.g., an oligosymptomatic infected relative or healthcare worker) is possible. This outbreak investigation illustrates that a molecular characterization is highly valuable to elucidate the potential transmission pathways in RSV outbreaks.

We followed the ORION statement for outbreak reporting [18] in many points in this report. However, we did not focus on evaluating the economic impact of the outbreak here (for instance due to reduced bed capacity because of isolation measures). Moreover, the prophylactic PCR-based screening we used is a resource-intensive measure, and we are aware that this might not be available in other settings.

## Conclusions

Although the importance of prevention and rapid containment of RSV outbreaks in hematology is well known and mostly comprehensive infection control measures are in place, transmissions may occur. We therefore underline that existing measures must be enforced by intensive training sessions for the wards’ staff and the patients’ visitors. Furthermore, prophylactic RSV screening is crucial for outbreak containment and even prevention. Integration of molecular analyses, such as high-resolution whole-genome sequencing, with classical epidemiological information on temporal and spatial events, can be instrumental in resolving outbreaks. The infection control management described here is—beyond RSV—essential in the COVID-19 pandemic.

## Supplementary Information

**Additional file 1.** GenBank accession numbers of RSV-A genomes applied in probe design

**Additional file 2.** GenBank accession numbers of RSV-B genomes applied in probe design

**Additional file 3.** Workflow of probe design pipeline

**Additional file 4.** Fasta files of all RSV capture probes

**Additional file 5.** Summary of short read sequencing results

**Additional file 6.** Coverage profiles of the RSV sequences obtained by target capture probe sequencing

**Additional file 7.** Overview of SNPs of all RSV sequences obtained

## Data Availability

The datasets generated during and/or analyzed during the current study are available from the corresponding authors on reasonable request. Patient data used in this study is confidential according to the German data privacy act, the ethics committee and the data protection commissioner of the Hannover Medical School. To protect patient confidentiality and participant’s privacy, data used for this study can be obtained in anonymous form only according to the data privacy act.

## Abbreviations

RSV: Respiratory syncytial virus
URTI: Upper respiratory tract infection
LRTI: Lower respiratory tract infection
COVID-19: Coronavirus disease 2019
RT-PCR: Reverse transcription polymerase chain reaction
HCWs: Health care workers
