# Supplementary material for: Target capture sequencing reveals a monoclonal outbreak of respiratory syncytial virus B infections among adult hematologic patients
Source: Antimicrob Resist Infect Control. 2022 Jun 21;11:88. doi: 10.1186/s13756-022-01120-z (PMC9210056; doi:10.1186/s13756-022-01120-z)
Supplement: Supplementary file 3 — Additional file 3. Workflow of probe design pipeline [file 13756_2022_1120_MOESM3_ESM.pptx]

## Slide 1
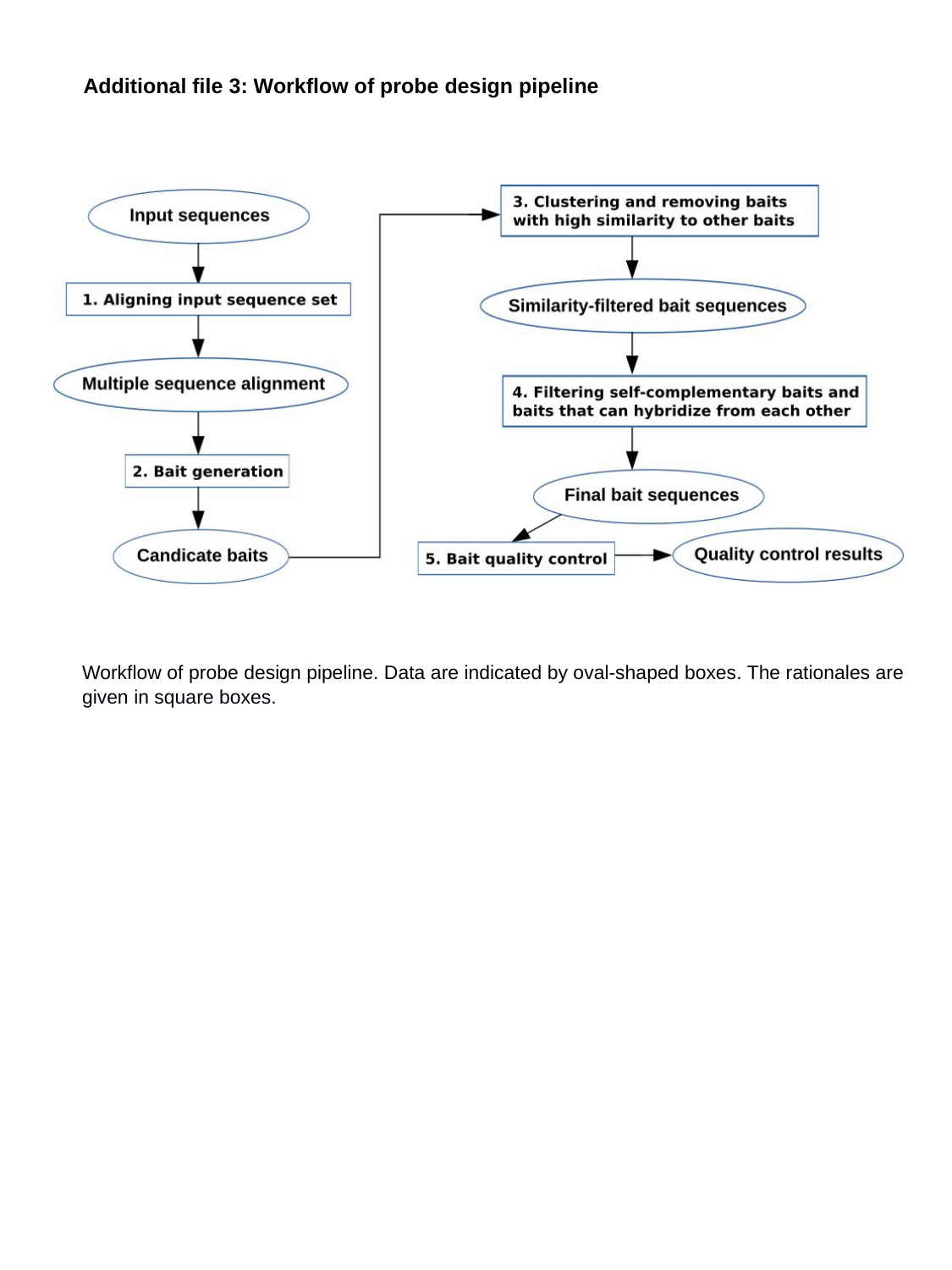

Additional file 3: Workflow of probe design pipeline
Workflow of probe design pipeline. Data are indicated by oval-shaped boxes. The rationales are given in square boxes.
