# Supplementary material for: Target capture sequencing reveals a monoclonal outbreak of respiratory syncytial virus B infections among adult hematologic patients
Source: Antimicrob Resist Infect Control. 2022 Jun 21;11:88. doi: 10.1186/s13756-022-01120-z (PMC9210056; doi:10.1186/s13756-022-01120-z)
Supplement: Supplementary file 6 — Additional file 6. Coverage profiles of the RSV sequences obtained by target capture probe sequencing [file 13756_2022_1120_MOESM6_ESM.pptx]

## Slide 1
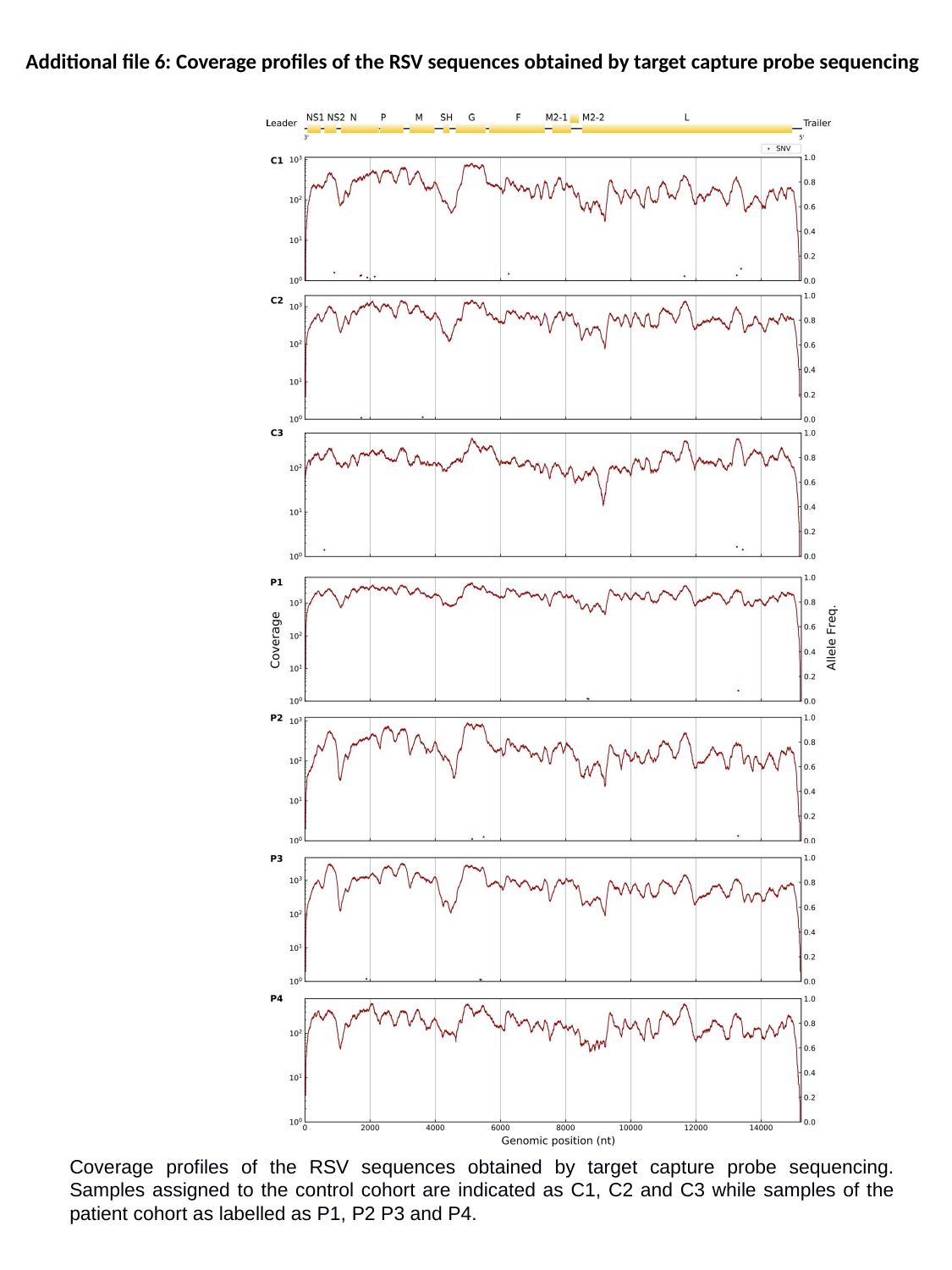

Additional file 6: Coverage profiles of the RSV sequences obtained by target capture probe sequencing
Coverage profiles of the RSV sequences obtained by target capture probe sequencing. Samples assigned to the control cohort are indicated as C1, C2 and C3 while samples of the patient cohort as labelled as P1, P2 P3 and P4.
